# Treatment of Uterine Tumors

**Published:** 1865-03

**Authors:** Wm. H. Byford

**Affiliations:** Prof. of Obstet., &c., in Chicago Medical College


### CHICAGO MEDICAL EXAMINER.

N. S. DAVIS, M.D., EDITOR.

VOL. VI. MARCH, 1865. NO. 3.

### Original Contributions.

### ARTICLE VII.

### TREATMENT OF UTERINE TUMORS.

BY WM. H. BYFORD, A.M., M.D., Prof. of Obstet., &c., Chic. Med. Col.

The surgical treatment of uterine tumors is always difficult, sometimes dangerous, and often fruitless in its results. The inacessible position of them, their proximity to important organs, and the want of proper knowledge of them, may account for these facts. Anything, therefore, that gives reasonable hopes of simplifying their cure, should commend itself to the attention of the profession. Of the modes now used, two seem to occupy the attention of the profession almost exclusively. One mode is to cut and extract them wholly from their bed in the tissue, and the other is to partially remove or destroy them, and depend upon disorganizing inflammation to finish the process of removal. A simpler method is to excite inflammation in them and depend upon it for their disintegration and elimination. This idea has been acted upon, and one case is reported by Prof. SIMPSON as being successful. It will be recollected that he punctured the investment of the tumor and slightly penetrated it with caustic potassa, which caused inflammation, sloughing, and evacuation of its substance. There is naturally a feeling of repugnance and fear in most minds against the use of this potent remedy for such purposes. I think an efficient and comparatively mild substitute for it, and one that gives less pain in its use, and which is not likely to spread inflammation to other tissues, and yet which excites this process where it is most needed, is found in the introduction of a foreign substance into the middle of the growth, and allow it there to remain until it excites disorganizing inflammation.

The mode of doing this is to insert a trochar into the substance of the tumor to its centre, when practicable, withdraw the stillette, pass the substance to be used through the tube, and hold it by means of a probe at the bottom of the perforation while the canula is withdrawn. In the course of a few days, inflammation will commence; disorganization, and after awhile, disintegration by suppuration and sloughing will cause the discharge of the substance of the growth.

The substance I have used is cotton wool; and I have in the two instances in which I have used it, previously to passing it through the canula, made fast to it a stout thread, so that it might be withdrawn if need be; but in neither case was it removed until washed away by the discharges. The tumor should be perforated through the vaginal or uterine canal, instead of through the abdominal wall.

Case I. Was a Mrs. G., aged 38 years, barren. She had been married twelve years. During the last four or five years she had been subject to more than the ordinary amount of sanguineous discharges. I was called to see her August 30th, 1863, when she informed me she had a polypus; that it had been removed once by a string and double canula, but in less than two months from the time of its removal it had, to all appearance, grown to be as large as it was before its removal. On making an examination, I found a tumor filling up a large distended uterus and implanted on its fundus, with no appearance of neck. It was nearly as large as a goose egg. After a few days, I removed as much as I could, which must have been two-thirds of it, with the ecrasseur. By the 1st of November, it seemed to have attained its former size. An examination of the portion removed showed it to be a specimen of the fibrous variety of polypi. On the 1st November, I plunged a trochar into the centre of it, to the distance of one and a-half inches, and withdrew the stillette, leaving the canula in the tumor. I next secured a piece of cotton wool, by tightly tying a stout thread to it, oiled it, and passed it down to the bottom of the canula, and holding it firmly in position by a strong probe, left it in as I withdrew the canula over it. The cotton remained in its place for ten days or two weeks, when it came away with the fetid discharge that had before commenced. I felt tempted to reintroduce it, but the whole polypus was so flabby, and in a state of such decomposition, that I did not do it. In three months from the introduction of the cotton, the tumor had entirely disappeared, and my patient remains well. She has some leuchorrhea and tolerably profuse menstruation. The only local treatment used was soapsuds injections several times a-day, as a matter of cleanliness. During the first six weeks after the operation, the patient was troubled with considerable diarrhœa, pain, and slight fever, but was not seriously ill.

CASE II. Was that of Miss E., aged 29 years. She had been subject to severe hemorrhages at menstrual periods, and for two years they had become alarmingly copious, and she had been debilitated, despondent, and nervous. She was very anxious to be relieved, as she desired to marry. An examination revealed an intramural fibrous tumor, occupying the anterior portion of the uterus, apparently about the size of a hen's egg. It extended very low, so as to encroach upon the vaginal portion of the cervix anteriorly. I thought it a good opportunity to try the cotton plug, and, accordingly, used it in the following manner. After introducing a catheter into the bladder and drawing off the urine, and a probe into the cavity of the uterus, to be sure of the direction with reference to the bulk of the tumor, I passed a rather small trochar into the middle of the tumor from the vagina upwards, and through the canula a piece of cotton secured by a thread, and left it *in situ*, and directed the patient to remain quiet until I saw her again.

In about four hours, I was requested to see her, as there was slight hemorrhage, and she was very much alarmed about it. The patient was so much alarmed that I determined, with a view to insure inflammation in the tumor, to pass some more cotton in the track of the canula. This I did through a speculum, with a stout probe, until it seemed pretty well filled up. Nothing further occurred, worthy of note, until about the fourth day. The patient then became feverish, and complained of pain, tenderness, and heat in the vagina. The parts were dry and hot; the pulse was about 100; the tongue white and dry, and there was some thirst. This moderate febrile reaction kept up for ten days, or two weeks, with varying degrees of intensity. During this time, about the sixth day, a sero-purulent discharge from the vagina began. It became more copious, and grew offensive; and on the tenth day, some of the cotton passed. This offensive discharge and feverish indisposition continued for about four weeks, and then slowly subsided. During this time, the treatment consisted of vaginal injections three times daily, quinine and sul. acid. There was so little inconvenience experienced in this case, that it seems hardly necessary to give a more circumstantial relation.

Other substances besides cotton may be used to excite inflammation in the tumor, and if sufficient destructive efforts do not follow the first introduction, a second might be attempted; and if still more irritation be desirable, the cotton might be dipped in tincture of cantharides, or other irritating fluid; or, following Dr. Simpson, a little caustic potassa passed through the canula and left in the tumor.

These cases and remarks are published with the view of encouraging a trial of some method similar to this, for the removal of tumors that cannot be taken out by the more summary process of excision. I think the treatment equally applicable to tumors elsewhere located, and of different composition; to any encysted tumor in which it is desirable to awaken disorganizing inflammation, and which is located in a place not accessible to the knife.

